# Measuring Setup for Experimental Research of Two-Coordinate Fiber-Optic Acceleration Sensors with Cylindrical Lenses

**DOI:** 10.3390/s22031125

**Published:** 2022-02-01

**Authors:** Elena Badeeva, Tatiana Murashkina, Andrey Motin, Saygid Uvaysov, Ainur Kozbakova, Daniel Sawicki

**Affiliations:** 1 Department of Radio Engineering and Radio Electronic Systems, Penza State University, 440026 Penza, Russia; badeeva_elena@mail.ru (E.B.); timurashkina@mail.ru (T.M.); 2 Institute of Physical Measurements, JSC “Scientific Research Institute for Physical Measurements”, 440026 Penza, Russia; motin.andrey92@yandex.ru; 3 Department of Design and Production of Radio-Electronic Means, Russian Technological University, 119454 Moscow, Russia; uvajsov@mirea.ru; 4 Laboratory of Artificial Intelligence and Robotics, Institute of Information and Computational Technologies CS MES RK, Almaty 050010, Kazakhstan; ainur79@mail.ru; 5 Department of Information Technologies, Almaty Technological University, Almaty 050013, Kazakhstan; 6 Departament of Electronics and Informational Technology, Lublin University of Technology, Nadbystrzycka 38d, 20-618 Lublin, Poland

**Keywords:** two-coordinate fiber-optic acceleration sensor, H-shaped elastic element, cylindrical lens, experimental setup, information conversion unit

## Abstract

The article presents the possibilities of using fiber-optic acceleration (FOC) sensors on products of rocket-space and aviation technology as part of information-measuring systems. A special measuring device has been developed for experimental confirmation of the main characteristics of the technical characteristics of the developed, two-coordinate fiber-optic acceleration sensors. The developed measuring setup for the experimental research of a two-coordinate fiber-optic acceleration sensor with two, cylindrical lenses fixed on two H-shaped elastic elements deflected under the influence of acceleration in two mutually perpendicular directions X and Y, intended for operation in harsh conditions of rocket and space technology. The experimental equipment consists of the developed setup for setting micromovements and an information conversion unit, including modules for signal conversion, transmission, power supply, signal amplification, and indication. Experimental dependences of the output voltage from the information conversion unit’s output on the micro-displacement in the range corresponding to the micro-displacements of the inertial mass with a cylindrical lens under acceleration in the range of ±100 m/s^2^ were obtained on the micro-displacement setting unit. The maximum value of the linearity error of the prototype acceleration sensor together with the information conversion unit was 0.07%. The conversion sensitivity of a two-coordinate fiber-optic acceleration sensor per the experimental dependences obtained on the Data Physics LE-612 MST/DSA 10–40 k vibration stand when exposed to sinusoidal vibration with an acceleration amplitude from 2 to 10 g in the frequency range from 5 to 2560 Hz was, on average, 3 mV/m/s^2^. The conducted experimental research confirms the performance of experimental samples of fiber-optic acceleration sensors together with an information conversion unit, as well as the achievement of high metrological characteristics.

## 1. Introduction

Sensors as a means of measuring physical quantities as part of information-measuring systems (IMS) for rocket-space and aviation technology (RS and AT) determine the quality of their functioning. They must be operable under the influence of external factors acting during the launch of spacecraft, with prolonged exposure parameters of outer space [1,2].

Measurements of accelerations onboard aircraft take up to 30% of the total number of all measurements [3,4]. Therefore, the creation of fiber-optic acceleration sensors (FOAS) for fiber-optic IMS (FOIMS) with improved technical characteristics is a complex scientific task [5,6]. The development of this science-intensive area has been identified as a priority for the development of the global aerospace industry. NASA, EKA, JAXA, and space agencies of the BRICS states have purposefully identified this direction in the national programs for the creation of advanced space systems and complexes for various purposes as promising [7,8].

FOAS, as part of FOIMS, has high mechanical strength, small dimensions, simple design, and, therefore, high reliability. They are chemically inert and made of dielectric materials, which ensures that there are no paths for an electric current to pass through them. In addition, they are highly resistant to high temperatures, mechanical shock, and other environmental influences. They allow remote measurements in hard-to-reach places.

Fiber-optic acceleration sensors differ from “electrical” sensors in that the measuring unit is an optical-mechanical device that does not contain electronic components, and the transformation of the measured value is carried out based on optical phenomena [9]. The division of FOAS into an optical-mechanical measuring unit and an optoelectronic unit is connected using a fiber-optic cable (FOC). This design makes it possible to carry the optoelectronic unit at a distance from several meters to a kilometer, allowing them to be used in conditions of radiation, spark-explosion-fire hazard, etc. [10,11,12].

Measurements can be carried out in extremely harsh environmental conditions such as exposure to high temperatures, high levels of electromagnetic interference, and environments of fire and explosion, providing galvanic isolation. Optoelectronic units can be in the immediate vicinity of the recording device of the telemechanics or control system. This makes it possible to create electrical channels for transmitting analog and digital signals, which ensures the fullest use of all the functions and capabilities of the information and measurement system.

Sensors of this type can be installed in the required number. Moreover, all of them are manufactured using the same technology and are unified in their schematic and technical solutions.

Using multichannel schemes for converting measuring signals and carrying out multiparametric measurements using several FOASs that are simple in schematic and technical design on the change in the intensity of the optical signal under the influence of the measured acceleration increases measurement accuracy, determining the feasibility of their use in promising FOIMS samples [13].

The analysis of the fiber-optic acceleration sensors on the market, both Russian and foreign, showed that an overwhelming majority of them are based on fiber Bragg gratings [14,15,16,17]. Less represented are fiber-optic acceleration sensors of the interferometric principle of converting an optical signal [18,19].

The main disadvantages of sensors based on both the Bragg grating and the interferometric type include the following:

1. Rapid failure of an optical fiber due to dynamic loads on the fiber, which in-turn leads to a breakdown of the device;
  Under the influence of increased shock loads, there is a possibility of instantaneous breakage of the optical fiber;
2. When using polymer optical fibers, it is possible for the fiber to become opaque as a result of radiation exposure, as well as physically deteriorating the fiber when exposed to corrosive agents (for example, fuel components). When using metalized fiber protection, the overall and mass characteristics of the devices are significantly increased;
3. The use of expensive devices for secondary information processing.

The disadvantages of sensors based on the use of Bragg gratings include technological difficulties in production, such as difficulties with aligning the optical systems of devices [20]. Sensors with such fibers have a higher cost than sensors using straight silica fibers.

**Figure 1:**
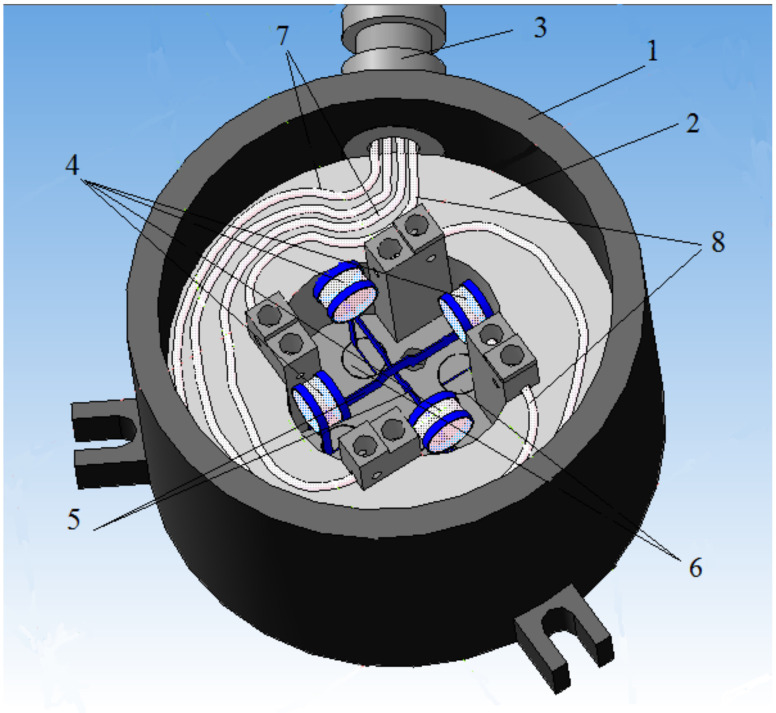
3D-model of two-coordinate FOAS. 1—body, 2—base, 3—fitting, 4—optically modulating element-cylindrical lens, 5—elastic element, 6—adjustment unit, 7—optical fibers of the X axes, 8—optical fibers of the Y axes.

**Figure 2:**
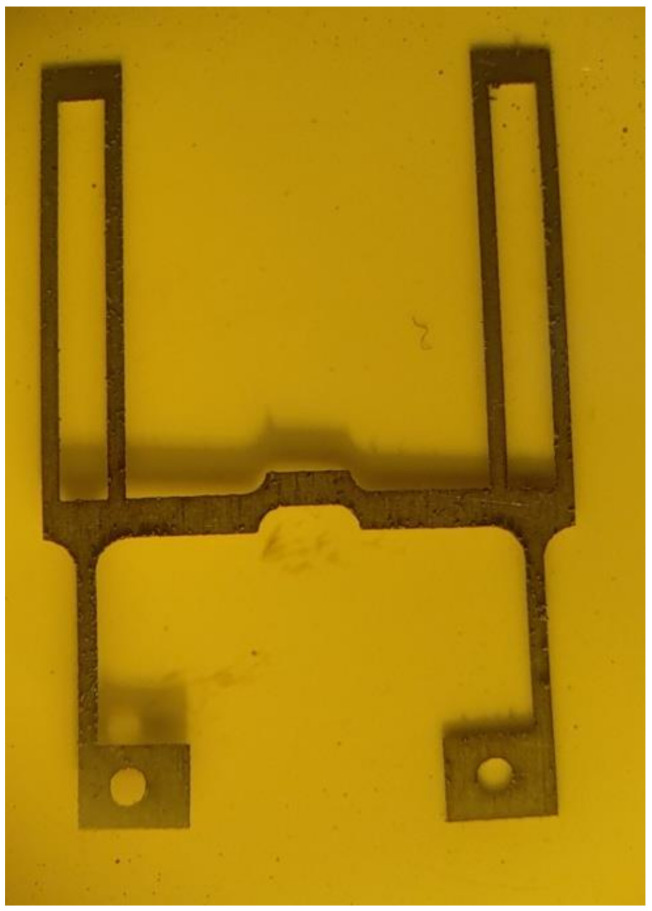
H-shaped elastic element.

Due to the rather complicated manufacturing technology of interferometric sensors and complex adjustment and alignment procedures, the main disadvantage of this type of sensor is the final price of the finished product. The indicated drawbacks limit the scope of application of FOAS on the market, in particular, making it difficult to use on aviation and rocket and space technology. In view of the constant growth in the number and variety of tasks solved by the acceleration sensors, it is necessary to constantly improve the existing measuring instruments and create new ones that meet the modern level of development for rocket-space and aviation technology. To reduce possible additional FOAS errors in difficult operating conditions and simultaneously expand their functionality by measuring accelerations in two directions, a two-coordinate acceleration sensor was proposed in [21,22,23,24] (Figure 1). In two-coordinate FOAS, two cylindrical lenses are fixed on two H-shaped elastic elements (Figure 2) deflected by an acceleration in two mutually perpendicular directions X and Y.

## 2. Experimental Methodologies

**Figure 3:**
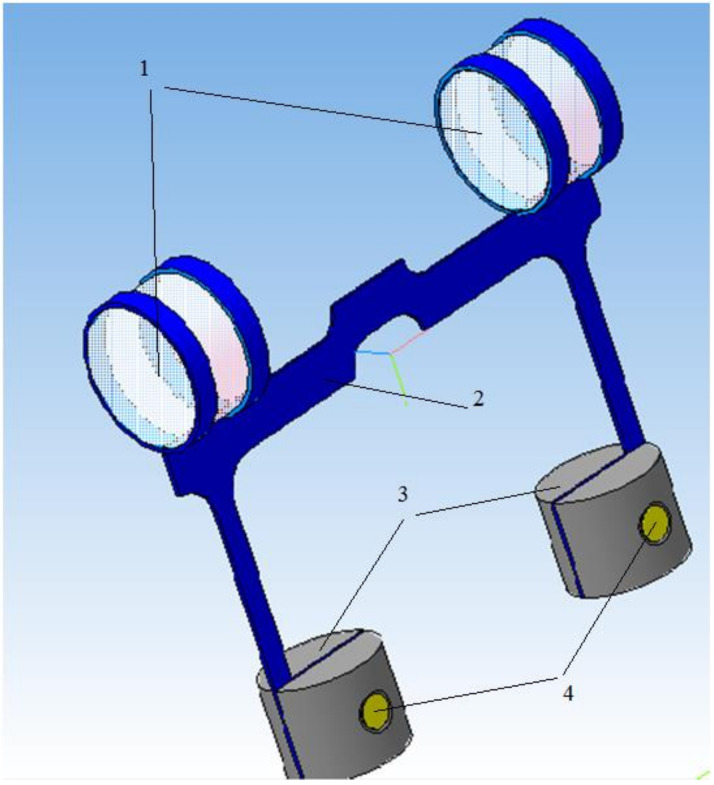
3D model of the sensing element. 1—cylindrical lens; 2—plane-parallel H-shaped spring, 3—adjustment unit, 4—pin.

The developed fiber-optic acceleration sensor contains a body 1 with a cover (not shown in Figure 2), in which base 2 is rigidly mounted [25]. On the base, there are two sensitive elements for measuring acceleration along two orthogonal planes (Figure 3), fixed on it through alignment unit 11, four supply optical fibers 8 and 9, laid directly on the base 2, and fixed in front of the optical element 4 at the base using micro screw 14 for even illumination and increased illumination.

In this case, the optical fibers of the two measuring channels are located relative to the optical elements 4 so that, in the static state of the FOAS, the light beams half overlap the ends of the optical fibers, and the optical fibers are laid on the base 2 so that their bending radius exceeds the minimum allowable.

The sensing elements consist of two elastic elements 5 in the form of plane-parallel H-shaped springs (see Figure 1), at the upper ends, two inertial masses are fixed in the form of optical elements 4 (see Figure 3). Optical elements 4 are transparent cylindrical lenses. Each optical element 4 is fixed to the elastic element 5 by means of two metal rings, which are wrapped around the optical element 4 and pulse-welded to the elastic element 5.

The springs are located perpendicular to each other and have grooves in the middle of the jumper strap for smooth passage of each other. Moreover, on the jumper strap of the elastic element of the X-axis, the groove is in the center of the lower part in order to implement the unimpeded movement of the elastic element of the Y-axis. In-turn, a groove is also on the jumper strap of the elastic element of the Y-axis but in the upper part.

The lower ends of the two sensitive elements 2 are fixed in four adjustment units 3 and are rigidly fastened with a pin 4 with a flaring. Adjustment units 3 include a cylinder divided into two parts for attaching an elastic element 2.

**Figure 4:**
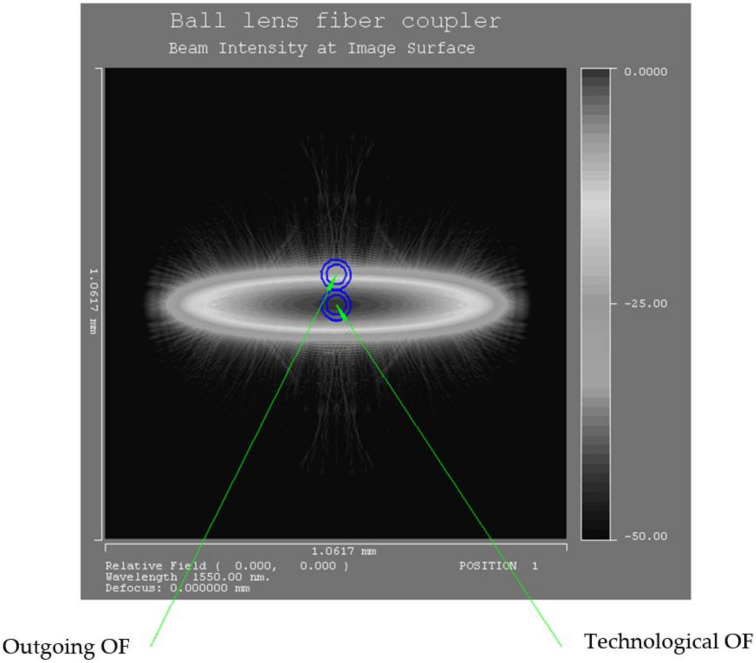
A light spot in the form of an oval, which is formed by two ellipses in the plane of the outgoing optical fibers.

When the light flux passes from the end of the feeding optical fiber through a cylindrical lens in the plane of the outgoing optical fibers, an oval-shaped light spot is formed by two ellipses (Figure 4) [13].

To achieve high sensitivity in converting the optical signal in the area of perception of the measuring information of the sensor, the geometric parameters of the sensor unit were determined. Mathematical modeling was carried out in the MatLab program.

**Figure 5:**
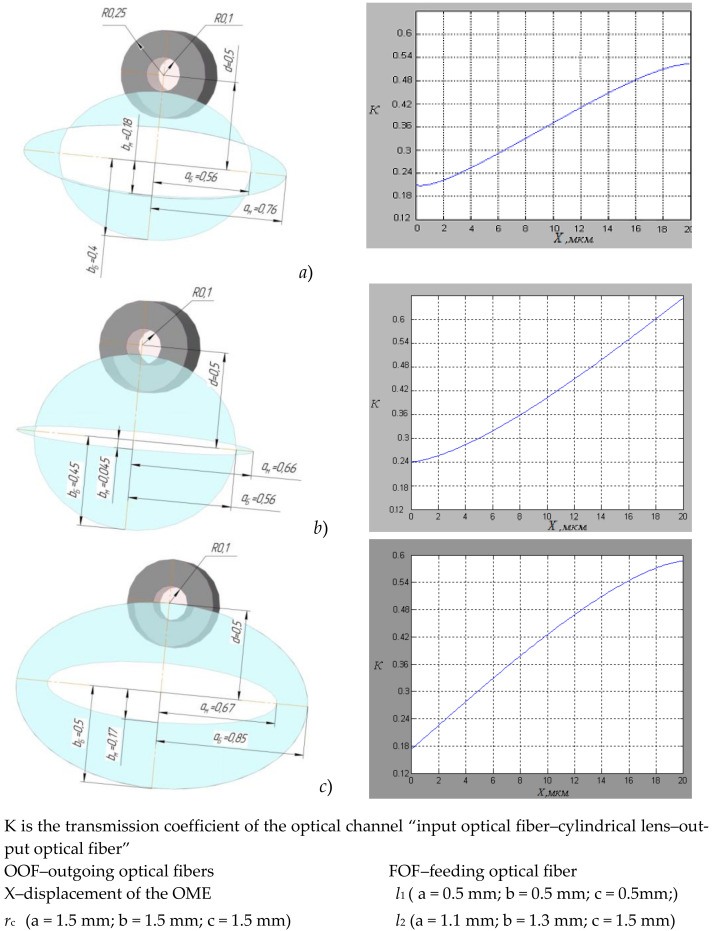
The main results of modeling an optical system at various variations of the distances “input optical fiber – cylindrical lens – output optical fiber”, (**a**) *l*_2_ = 1.1 mm, (**b**) *l*_2_ = 1.3 mm, (**c**) *l*_2_ = 1.5 mm.

Several shapes of light spots were modeled in the plane of the outgoing optical fibers, OOF, and the functions of the change in intensity of the light flux corresponding to the indicated forms with variations in the distance *l*_1_ from the end of the feeding optical fiber, FOF, to the lens surface and *l*_2_ from the lens surface to the receiving ends of the OOF, as well as the lens radius r_c_ (Figure 5).

The simulation results made it possible to choose the most optimal FOAS parameters. The task is to confirm the technical characteristics of new sensors by conducting experimental studies. The goal of the work is to develop a measuring setup for experimental research of a two-coordinate fiber-optic acceleration sensor with two cylindrical lenses and to determine its main metrological characteristics.

**Figure 6:**
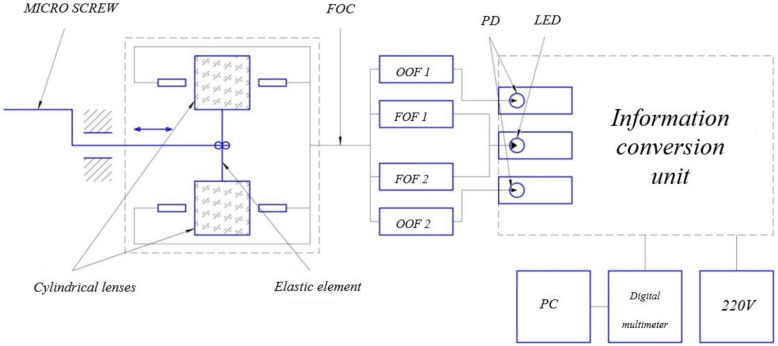
Installation for setting micromovements with ICU. PD—photodiode; SD—LED; PC—personal computer; FOC—fiber optic cable.

To carry out experimental studies to confirm the performance of the developed FOAS, an experimental setup is used, which consists of an installation for setting micromovements (ISMM) and an information conversion unit (ICU) (see Figure 6).

ISMM includes a 9S65M calibration micro-screw (scale division—1 micron), which is fixed on the rack of the experimental setup using an expansion grip. With the help of a calibration micro-screw, the micro-displacement of the elastic element and, accordingly, the cylindrical lenses fixed on it along the X or Y-axis parallel to the optical axis of the optical fibers is set. A USB microscope mounted on a fixed base of the experimental setup is used to position cylindrical lenses with high accuracy. When the lens is moved in the plane of the outgoing optical fibers (OOF), the position of the light spot (hollow ellipse) formed by the cylindrical lens changes [23].

**Figure 7:**
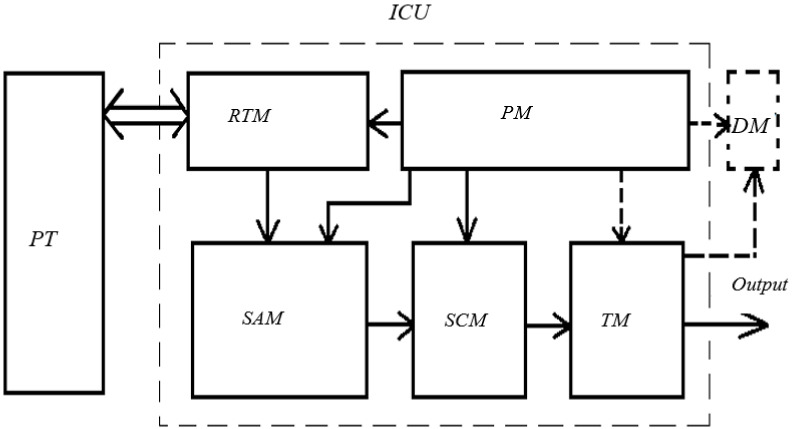
Block diagram of a modular block for converting information. ICU—information conversion unit; SCM—signal conversion module; PT—primary measuring transducer; TM—transmitting module (if necessary); RTM—receiving and transmitting module; PM—power module; SAM—signal amplification module; DM—display module (if necessary).

ICU is made in a modular design with the following capabilities [24]:

1. Amplification of a low input signal;
2. Suppression of noise and electrostatic interference;
3. Presentation of information in the form of various types of the output signals (current signal, output signal in the form of voltage, output signal via RS425 and USB channels, direct visualization of the output signal on the ICU indicator display). ICU has an output signal range in the form of a constant voltage (0…6) V. The satisfaction of these requirements is possible due to the division of the main parts of the ICU into replaceable units, which makes it possible to use it both as a part of a laboratory stand and in a critical part of a recording device without changing the layout of printed circuit boards, replacing radio elements, etc. The block diagram of such an ICU is shown in Figure 7.

**Figure 8:**
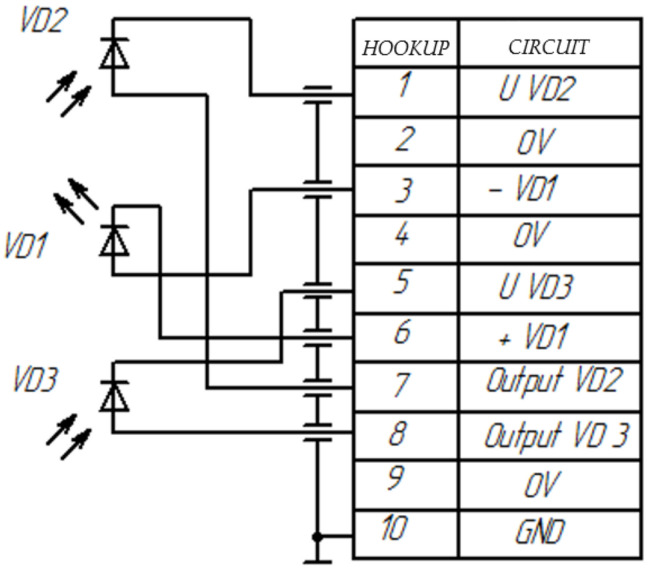
Functional diagram of the receiving and transmitting module RTM. VD1—LED; VD2, VD3—photodiodes.

The receiving and transmitting module (RTM) is a radiation source (RS) combined in the main body (for example, 3L107B LEDs), and radiation receivers (RR) (for example, PD265 photodiodes) have the ability to connect them from the side of the emitting and receiving areas with optical fibers, and from the side of electrical outlets—with an electrical lead (Figure 8). In this case, photodiodes can operate in three modes: photodiode, short circuit mode, and photovoltaic [25]. Due to the low value of the output signals of the photodiodes [26], it is imperative to ground the conductors and shield the RTM main body to prevent the influence of interference on the measurement results (in the prototype ICU, power and signal lines in the form of a twisted pair are used).

By means of an electrical outlet, the RTM is connected to the signal amplification module of the SAM and the power supply module of the PM.

### 2.1. Selection of Radiation Sources and Receivers

To ensure reliable operation of FOAS, radiation sources must meet the following requirements:

1. The design of the RS should provide a high radiation power;
2. The radiation wavelength of the RS should coincide with the minimum spectral losses of optical fibers;
3. The spectral range of the RS should be consistent with the range of the maximum sensitivity of the RR;
4. The directional diagram of the RS should be circular and evenly distributed;
5. RS must have a minimum temperature coefficient of the wavelength of the maximum spectral distribution (0.05 nm/°C) and maintain performance over a wide temperature range;
6. The principle of building RS should provide maximum performance;
7. Overall dimensions, weight, and power consumption of the RS should be as small as possible;
8. The source must have high reliability and long service life (equal to or greater than the service life of the measuring instrument in which it is used).

Implementation of FOAS with RS operating in the infrared range has the best characteristics [27,28].

Such sources have advantages that are relevant for products of rocket-space and aviation technology:

1. Low power consumption (high efficiency);
2. The ability to work in both pulsed and continuous modes;
3. A wide range of pump currents and modulation frequencies (compared to existing LED counterparts, applied design solutions ensure more than a 3-fold increase in the radiation power and the ability to operate in a continuous mode up to pump currents of 200 mA);
4. Concentration of radiation in a narrow-angle (up to 15 degrees);
5. Explosion safety (the heating of the diode does not exceed 20 degrees against 750 or more degrees for thermal radiation sources);
6. High speed (modulation reaches 100 MHz);
7. Service life up to 75,000 hours;
8. A wide range of operating temperatures (from minus 50 to +60 °C);
9. Small dimensions;
10. Ample opportunities for adjusting the radiation parameters to the individual requirements of the customer;
11. “Relatively low” cost (in comparison with the best foreign counterparts with similar technical characteristics).

The most reliable and widespread sources of infrared radiation are light-emitting diodes of the type 3L107 A, B, 3L108 A, 3L119 A, B, the spectral range of radiation of which lies in the range 0.94…1.21 μm, having sufficient radiation power (10…40) megawatt, high service life (at least 50,000 h), small dimensions, and efficient under mechanical and climatic influences.

In the developed FOAS, radiation receivers (RR) perform the function of receiving and converting optical radiation into electrical radiation, which is then amplified and processed in electronic circuits. The choice of RR must be performed based on the condition of maximum spectral matching with the characteristic of the RS.

RR must meet the following requirements

1. To have temperature and time stability of characteristics, maximum sensitivity, low response time;
2. Have the ability to detect both pulsed and continuous signals (in particular, compared to photoresistors, photodiodes have the ability to detect continuous radiation, do not require a power source, and have more than tenfold superiority in sensitivity and speed);
3. Provide a small angle of view (up to 15 degrees);
4. Have low weight and dimensions, accurately reproduce the shape of the received signal.

The most reliable in harsh operating conditions are semiconductor photodiodes (PD), which satisfy all of the above requirements to the greatest extent [29].

P–i–n-diodes are used with sufficient optical power of the incoming signals. At the low power of the input optical signal, avalanche PDs are used. Compared to p–i–n-diodes, avalanche PDs have a much higher bias voltage and a more complex structure. Since the avalanche multiplication process is statistical, additional noise appears. In addition, in order to make the avalanche multiplication factor more constant, it is necessary to resort to temperature and supply voltage stabilization. Series-produced p–i–n and avalanche PDs based on Si are almost ideal RRs, however, their spectral characteristics are limited to the range of 0.5…1.0 µm.

For the spectral range of 1.1…1.6 μm, germanium APDs are used, as well as p–i–n and APDs based on JnGaAs, GaAlSb, and JnGaAsP. Simpler and cheaper but less sensitive p–i–n diodes are mainly used in systems where the optical signal is transmitted over short distances up to 100 m, if, over long distances, it is advisable to use more complex and sensitive APDs.

**Figure 9:**
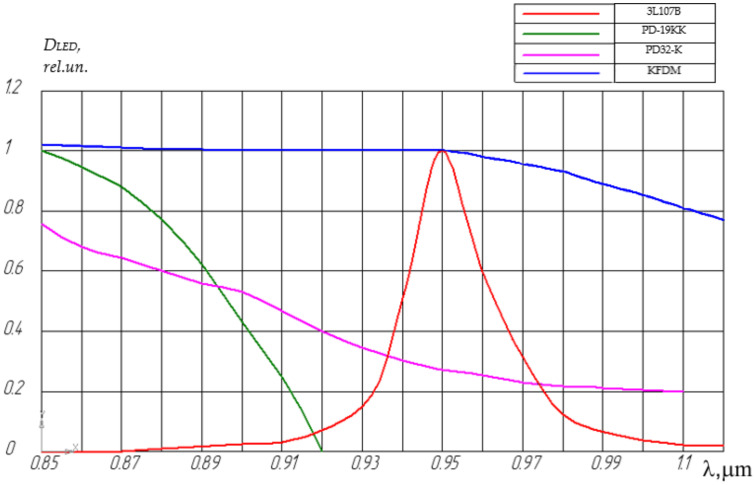
Comparison of LED 3L107B spectral emission and sensitivity of chosen photodiodes.

Figure 9 shows the spectral characteristics of the infrared LED 3L107B and photodiodes KFDM and PD-19KK [30]. Analysis of these characteristics showed that the spectral range of the PD-19KK photodiode partially coincides with the spectrum of the 3L107B LED, and the spectral matching coefficient is η(λ) ≈ 0.5. The best match for the 3L107B LED in terms of the spectral matching coefficient can be considered the KFDM photodiode; the spectral sensitivity range completely covers the emission range of the 3L119B LED. In this case, η(λ) ≈ 1.

In the developed FOAS, photodiodes of the KFDM, PD-19KK, PD-32K, and PD-20K types are used, the maximum sensitivity of which is at a wavelength of approximately 0.95 μm.

### 2.2. Signal Amplification Module (SAM)

**Figure 10:**
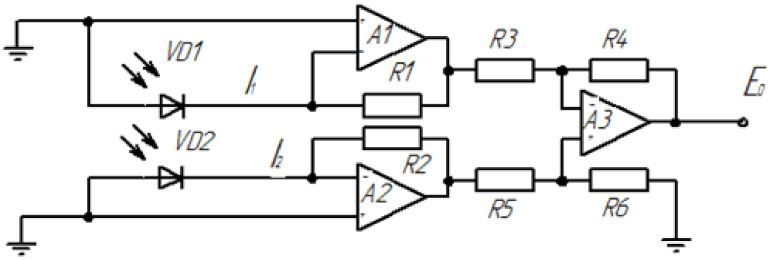
Simplified functional electrical diagram of the SAM signal amplification module.

A simplified functional diagram of the SAM is shown in Figure 10.

To the input of each “current-voltage” converter, A1 and A2 are connected via a separate diode, VD1 and VD2, from the signal transmission-reception module. Their currents I1 and I2 generate independent voltages at outputs A1 and A2, after which they pass through the differential amplifier A3 to eliminate the common-mode component.

It is worth noting that the resistances are R1 = R2, and R3 = R4 = R5 = R6.

The remaining output voltage E0 is proportional to the difference between the two photocurrents as a measure of the relative illumination.

Special attention is paid to the immunity of the interference of this module.

Electrostatic and magnetic noise coupling is reduced by using differential photodiode switching circuitry. In this case, communication occurs through mutual inductance; therefore, when designing this module, the dimensions of the conductor loops are minimized; it is also advisable to use the module shielding. However, the influence of magnetic noise coupling is not eliminated by the electrostatic shield. Therefore it is necessary to apply suppression of interference at its source. For this, the power supply of the signal amplification module must be removed at a sufficient distance. Accordingly, the use of a modular ICU has the advantage of allowing separate shielding for the power supply module so that most of the magnetic fields remain inside the power circuit converters. The implementation of the SCM signal conversion and TM information transmission modules in separate shielded enclosures has its advantages, as in this case, radio frequency interference on the SAM signal amplification module is reduced.

At high frequencies, operating amplifiers have little gain and little common-mode rejection, and therefore, cannot reject radio frequency signals. Because of these operating amplifier limitations and bandwidth limitations in the main circuit of the “current-voltage” converter, the signals under investigation cannot be in the radio frequency range. Filtering can be used to remove unwanted signals if it can be applied to the amplifier input. Post-amplifier filtering is less effective because the operating amplifier can act like a radio frequency detector separating lower frequencies from the carrier. Further reduction of these types of noise can be obtained by using radiofrequency shields and “ground” layers on the printed circuit board.

### 2.3. Signal Conversion Module (SCM)

The SCM is made in the form of a voltage converter of the SAM to the voltage of the output signal. Another feature of SCM is that they can be used to introduce additional functions of the ICU, for example, zero correction, etc. In the SCM, it is also necessary to apply shielding to eliminate the influence of interference on the operational amplifiers of the SAM.

#### Transmitting Module (TM)

The TM performs the function of transmitting a signal from the ICU for further processing. TM can be made in various types of execution:

1. When used in the form of an output signal of current or voltage, it is advisable to execute the TM in the form of a socket;
2. When using a digital output signal, you can use the output interfaces USB or RS425, etc. It is possible to use the radio frequency output signal through various communication channels;
3. It is possible to access the display of the indication module IM on the ICU itself, for example, if the device is used as a laboratory bench or in the field.

### 2.4. Power Module (PM)

**Figure 11:**
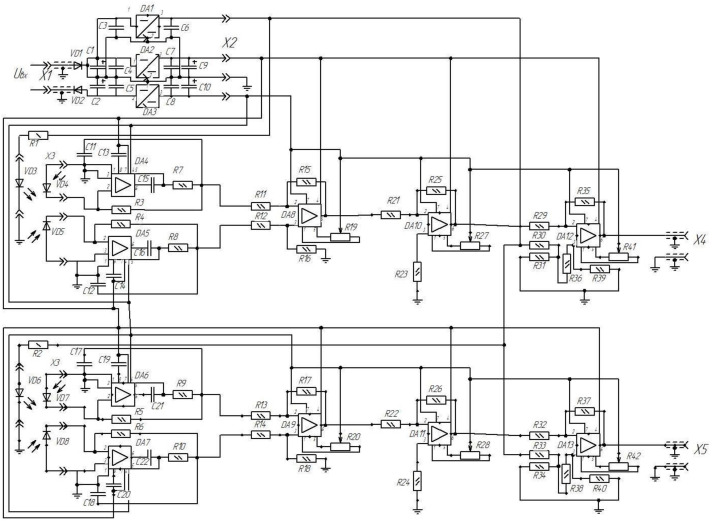
Basic electrical diagram of ICU.

The convenience of creating separate PMs is that it becomes possible to power the ICU from the available power sources. The prototype is powered by a 220 V variable-voltage transformer with subsequent rectification. A schematic electrical diagram of the ICU has been developed (Figure 11).

A constant voltage of 27 V is supplied to the input X1. Diodes VD1 and VD2 are used to protect the ICU circuit against incorrect supply polarization (and in the case of a prototype for converting alternating-current voltage to direct-current voltage). Electrolytic capacitors C1, C2, C9, and C10 are designed to form a bipolar supply voltage. Microcircuits DA1, DA2, DA3 are voltage stabilizers and form supply voltages +5 V, +15 V, minus 15 V, respectively. Ceramic capacitors C3–C8 are used according to the typical connection diagram for voltage converters.

The voltage through the X2 connector is supplied to the operating elements of the ICU (in the prototype, the X2 connector is not used; instead, a twisted pair is used for each voltage).

The +5 V supply voltage is supplied through the resistors R1, R2, respectively, to the LEDs VD3 (channel of the X-axis) and VD6 (channel of the Y-axis) through connector X3 (in the prototype by means of a twisted pair). For supply voltage +15 V, minus 15 V is supplied respectively to the power supply of operational amplifiers DA4–DA13.

The luminous flux, after passing through the conversion in the primary measuring transducer, is supplied to the photodiodes VD4–VD5 (channel of the X-axis) and VD7-D8 (channel of the Y-axis). Photodiode currents through connector X3 (in the prototype with a twisted pair) are fed to the inputs of precision current-voltage converters) DA4–DA5 (X-axis channel) and DA6–DA7 (Y-axis channel). The resistors and capacitors in the diagram are shown for a typical connection of specific operational amplifiers and, accordingly, can be replaced when using a different type of operational amplifier.

The voltages generated at the outputs of the current-voltage converters are fed to the inputs of the operational amplifiers DA8 (channel of the X-axis) and DA9 (channel of the Y-axis). In this case, they act as a voltage follower to generate a bipolar output signal. Accordingly, when one of the photodiodes of each measuring channel is illuminated and, accordingly, the light intensity decreases, either a positive or negative voltage is formed on the other photodiodes of each channel. The resistors shown in the diagram are for a typical wiring diagram. It should be noted that resistors R19 and R20 are used to balance the circuit, which greatly simplifies the adjustment, while increasing the accuracy and sensitivity of the circuit.

The voltage from the outputs of the operational amplifiers DA8 (channel of the X-axis) and DA9 (channel of the Y-axis) is fed to the inputs of the operational amplifiers DA10 and DA11. They have a similar connection scheme. However, the difference is that they amplify the signal by using large resistors R25 and R26. In the prototype, the signal is amplified to a value of minus 3 V and + 3 V. The use of balancing resistors R27 and R28 is optional but desirable for more accurate adjustment of the output signal.

The amplified voltage is applied to the inputs of the operational amplifiers DA12 (channel of the X-axis) and DA13 (channel of the Y-axis). They compare amplified photodiode signals with a reference voltage of +3 V. The reference voltage is formed from the + 5 V power supply circuit with voltage dividers consisting of resistors R30, R31, R33, and R34. In a static state, the voltage at outputs X4 and X5 will be equal to +3 V. When accelerated, in one case, it will increase to +6 V; in the other, it will decrease to 0 V.

**Figure 12:**
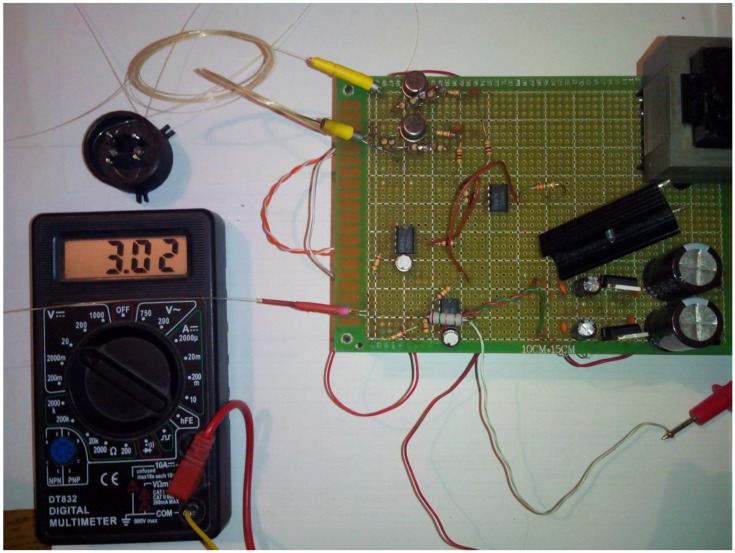
Mock-up experimental prototype FOIMS, including prototype FOAS connected to ICU.

The prototype experimental FOAS is connected to the prototype ICU without the use of connecting sockets and matching devices (Figure 12).

## 3. Results of Experimental Research

To demonstrate the efficiency of the developed FOAS, the cover of FOAS was removed, which introduced a small error in the measurement result. When the cover of the FOAS main body was closed, the output signal values were set to the required values of 0 V—at the beginning of the measuring range or 6 V—at the end of the measuring range.

**Figure 13:**
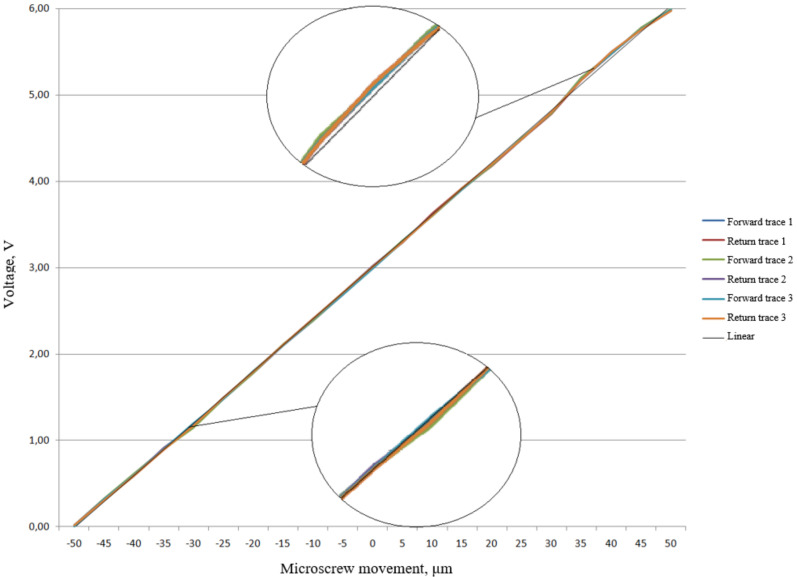
Dependence of the output signal from the ICU on the micro-displacement of the cylindrical lens.

Experimental dependences of the output voltage from the ICU output on the micro-displacement *U_exp_* = f (*x*) in the range corresponding to the—of the inertial mass with a cylindrical lens under acceleration in the range of ±100 m/s^2^ were obtained on the setup for setting the micro-displacement (Figure 13).

The calculated values of the cylindrical lens micro-displacements in the measuring range were ±50 µm [10]. U_exp_ output values were recorded every 5 µm in the forward and reverse directions. This operation was repeated three times.

An analysis of the averaged experimental dependence *U_exp_* = f (*x*) of the prototype FOAS together with the ICU showed that the maximum value of the linearity error |γ|_max_ is 0.07%. The optical signal conversion sensitivity averaged 0.06 V/μm.

FOAS tests were carried out on the vibration stand Data Physics LE-612 MST/DSA 10–40 k (equipped with an exemplary vibration sensor 8370 Bruel Kjaer). The output signal from the ICU was fed to the input of a Keysight 34401a multimeter with the output of the graphical dependence to a personal computer using the Agilent Technologies 34410a software installed.

FOAS was subjected to sinusoidal vibration with an acceleration amplitude of 2 to 10 g in a frequency range from 5 to 2560 Hz, smoothly increasing the frequency and amplitude of acceleration in a given range, and then smoothly decreasing, at a rate of no more than one octave per minute. The vibration exposure time was 15 minutes in the forward and reverse directions.

**Figure 14:**
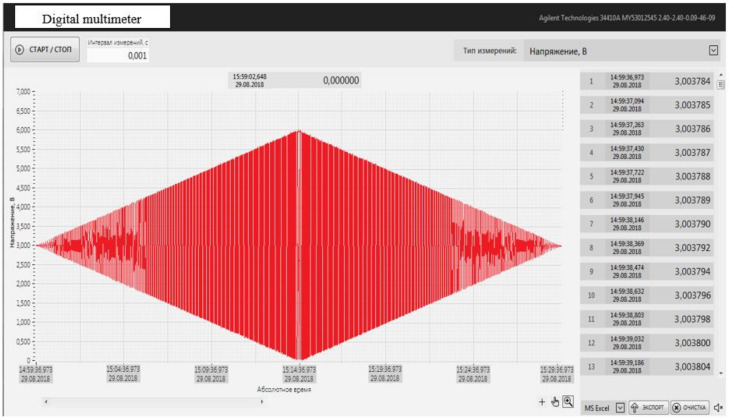
Data Physics LE-612 MST/DSA 10–40 k. Experimental dependences *U* = f (*g*) obtained on the vibrating test stand Data Physics LE-612 MST/DSA 10–40 k.

Similar tests were carried out in both the X and Y coordinates. The results are consistent in both directions. The dependency graph of the output signal *U* = f (*g*) is shown in Figure 14.

The sensitivity of the conversion of FOAS is on average 3 mV/m/s^2^.

## 4. Conclusions

The authors solved the problem of measuring accelerations onboard aircraft using two-coordinate fiber-optic acceleration sensors with improved technical characteristics. Original sensitive elements were used in the form of elastic elements with cylindrical lenses attached to them to simultaneously act as optically modulating and inertial elements, making it possible to significantly reduce the mass-dimensional characteristics of the sensor and simplify the installation of sensors at the measurement object.

The following provisions are new to the presented work:

1. A measuring device developed for experimental tests of a two-coordinate fiber-optic acceleration sensor;
2. For the first time, the micro-displacements of the cylindrical lenses along the X or Y-axis are set using a calibration micro-screw with a step of 5 microns, simulating their movement in relation to the ends of the optical fibers during vibrations;
3. A microscope was used for the first time to precisely position cylindrical lenses.

The conducted experimental research confirms the performance of the experimental samples of FOAS together with the electrons with the information conversion unit (ICU) and the achievement of high metrological characteristics.
